# A 24‐nt miR9560 modulates the transporter gene *BrpHMA2* expression in *Brassica parachinensis*

**DOI:** 10.1002/tpg2.70013

**Published:** 2025-03-19

**Authors:** Yongsheng Bai, Xiaoting Wang, Shahid Ali, Yang Liu, Jiannan Zhou, Meiting Liu, Shuai Liu, Yulin Tang

**Affiliations:** ^1^ Guangdong Provincial Key Laboratory for Plant Epigenetics, Guangdong Technology Research Center for Marine Algal Bioengineering, Shenzhen Public Service Platform of Collaborative Innovation for Marine Algae Industry, Longhua Institute of Innovative Biotechnology, College of Life Sciences and Oceanography Shenzhen University Shenzhen China; ^2^ Shaanxi Academy of Traditional Chinese Medicine Xi'an China; ^3^ Guangdong Academy of Forestry Guangzhou China; ^4^ Key Laboratory of Tropical Fruit Biology (Ministry of Agriculture), South Subtropical Crops Research Institute Chinese Academy of Tropical Agricultural Sciences Zhanjiang China

## Abstract

MicroRNAs (miRNAs) control gene expression in plant through transcript cleavage and translation inhibition. Recently, 24‐nt miRNAs have been shown to direct DNA methylation at target sites, regulating the neighboring gene expression. Our study focused on miR9560, a 24‐nt miRNA induced by cadmium (Cd) stress in *Brassica rapa* ssp. *parachinensis* (*B. parachinensis*). Phylogenetic analysis revealed miR9560 predominantly emerged in the Rosanae superorder and was conserved in Brassicaceae, with potential target sites adjacent to transporter family genes *HMAs*. RNA gel blotting showed that mature miR9560 was only detected in various *Brassica* crops roots after Cd stress. In *B. parachinensis*, miR9560's putative target site is upstream of *BrpHMA2*, an afflux‐type Cd transporter. In a transient expression system of *B. parachinensis* protoplasts, the expression of miR9560 increased the DNA methylation upstream of *BrpHMA2*, reducing the transcription of *BrpHMA2*. This regulation was also observed in Arabidopsis wild‐type protoplasts but not in the mutants *dcl234* and *ago4* with impairments in the RNA‐dependent DNA methylation (RdDM) pathway. We deduced that miR9560 modulates *BrpHMA2* expression via the RdDM pathway, potentially regulating Cd uptake and movement in *B. parachinensis*. Furthermore, this regulatory mechanism may extend to other *Brassica* plants. This study enhances our comprehension of 24‐nt miRNAs role in regulating Cd accumulation within *Brassica* plants.

## Core Ideas

- Phylogenetic analysis reveals miR9560 emerged in Rosanae and is conserved in Brassicaceae.
- Experimental proof shows that miR9560 is induced by cadmium (Cd) stress in a tissue‐specific pattern.
- miR9560 relies on the RNA‐directed DNA methylation pathway to regulate *BrpHMA2* under Cd stress.
- DNA methylation modulates *BrpHMA2* expression, enhancing Cd stress tolerance.

**Abbreviations**
Cd: cadmium
cmiRNAs: canonical miRNAs/21‐nt miRNAs
DAC: days after 50 µM Cd(NO_3_)_2_ treatment
EV: empty vector
HMA: the heavy metal ATPase
lmiRNAs: long miRNAs/24‐nt miRNAs
miRNAs: microRNAs
RdDM: RNA‐dependent DNA methylation
RT‐qPCR: real‐time quantitative reverse transcription polymerase chain reaction

## 1 INTRODUCTION

MicroRNAs (miRNAs) are non‐coding RNAs of 21–24 nucleotides (nt) that affect a variety of biological processes (Moran et al., 2017). Accumulating evidence suggests that miRNAs function as plant development regulators and that they respond to various environmental stimuli (Song et al., 2019; Sunkar & Zhu, 2004; Sunkar et al., 2006). Mature miRNAs may be classified into two categories based on their length: the 21‐nt miRNAs (canonical miRNAs, cmiRNAs) and the 24‐nt miRNAs (long miRNAs, lmiRNAs) (Arikit et al., 2013). Both types are transcribed by RNA polymerase II, but their subsequent processing and functions diverge. LmiRNAs are potentially generated by DCL3 and then incorporated into AGO4 to regulate DNA methylation at specific sites and thereby influence gene expression. In contrast, cmiRNAs are created by DCL1 and then incorporated into AGO1 and suppress the expression of their target genes by transcript cleavage or repression of translation (Arikit et al., 2013; Chen & Rechavi, 2022; Wu et al., 2010; Yu et al., 2019). Wu et al. (2010) first identified a group of lmiRNAs in rice and noted that these lmiRNAs were infrequently found in plants with lower levels of accumulation. Subsequent studies revealed that additional lmiRNAs, such as miR1873 (Zhou et al., 2020), miR1876 (Jiang et al., 2020), miR812w (Campo et al., 2021), and miR1871 (Y. Li et al., 2022), were capable of enhancing the methylation status of certain DNA sites to confer defense against bacterial and fungal infections. Plant DNA methylation occurs in different sequence contexts (CG, CHG, and CHH, where H stands for A, T, or C) primarily via the RNA‐dependent DNA methylation (RdDM) mechanism (Erdmann & Picard, 2020; Lister et al., 2008).

Cadmium (Cd) is an agricultural contaminant that is harmful to plants, causing damage to photosynthetic activity and membrane deformity and affecting plants' growth and productivity (Fu et al., 2019). Genome‐wide miRNA analyses have confirmed the roles of miRNAs in Cd stress in various plants, including *Oryza sativa* (Ding et al., 2013), *Brassica napus* (Fu et al., 2019), *Boehmeria nivea* L. (Chen et al., 2018), *Sedum alfredii* (Han et al., 2016), and *Solanum tuberosum* L. (Yang et al., 2021). These studies have identified numerous miRNAs, including miR156, miR164, miR172, miR408, miR858, and miR2111, that exhibit differential expression in response to Cd stress. Our previous small RNA‐Seq analyses revealed that several miRNAs, including miR403, miR169, miR159, miR397, and a 24‐nt lmiRNA, miR9560, were differentially expressed in *Brassica rapa* ssp. *parachinensis* (*B. parachinensis*) when the plants were exposed to Cd stress (Y. Liu et al., 2020). However, investigation of the function of Cd‐induced miRNAs, particularly the lmiRNAs, remains limited.

Our previous small RNA‐Seq analyses showed that a 24‐nt miRNA, miR9560, was induced in *B. parachinensis* by Cd stress (Y. Liu et al., 2020). This miRNA was initially detected in the small RNA‐seq of *Citrus apomixis* in 2016 (Taylor et al., 2017). To elucidate the function of miR9560 in *B. parachinensis*, we predicted its potential target site, which was identified upstream of *BrpHMA2* (heavy metal ATPase 2). *HMAs* belong to a subfamily of the P‐type ATPase superfamily, crucial for transporting heavy metals (i.e., Zn^2+^, Cu^2+^, and Cd^2+^) in plants such as *Arabidopsis thaliana* (Hussain et al., 2004; Wong & Cobbett, 2009), *Oryza sativa* (Satoh‐Nagasawa et al., 2012; Takahashi et al., 2012), *Triticum aestivum* (Tan et al., 2013), *Sedum plumbizincicola* (Zhao et al., 2019), and *Arachis hypogaea* (J. Li et al., 2024). Our previous study showed that *BrpHMA2* is co‐regulated by two transcription factors, BrpNAC895 and BrpABI449, and functions in Cd uptake and long‐distance transport in plants (S. Liu et al., 2022).

In the current study, phylogenetic analysis revealed that miR9560 predominantly emerged in the Rosanae superorder, and the identified potential target sites of miR9560 in some species are adjacent to *HMA* genes. The functions of miR9560 in regulating DNA methylation and gene transcriptional expression in *B. parachinensis* were comprehensively analyzed by using a transient gene expression system in protoplasts. A novel regulatory interaction between miR9560 and *BrpHMA2* was identified. We propose that miR9560 fine‐tunes the expression of *BrpHMA2* through the RdDM pathway, thereby playing a role in controlling Cd uptake in *B. parachinensis*. This mechanism may represent a common regulatory strategy within *Brassica* plants.

## 2 MATERIALS AND METHODS

### 2.1 Plant material and growth conditions

The seeds of *B. parachinensis* ‘Youlv 702 (YL)’ were obtained from the Vegetable Research Institute, Guangdong Academy of Agricultural Science. *Brassica carinata, B. juncea, B. chinensis*, oilseed rape *B. napus* ‘Westar’, and *B. napus* ‘ZS11’ seeds were obtained from Huazhong Agricultural University. *Brassica napus* ‘HY6’, *B. napus* ‘HY8’, and *B. napus* ‘YY9’ seeds were obtained from the Yunnan Academy of Agricultural Sciences. Seeds were surface‐sterilized by NaClO (0.4%–0.7%) and ethanol (75%), grown on ½ MS medium for 5 days, and then transferred to a simple hydroponic culture device with ½ Hoagland nutrient solution (pH 5.8–6.0). A final concentration of 50 µM Cd(NO_3_)_2_ was applied to the nutrient solution 7 days after transplantation for heavy metal treatment. The *Arabidopsis thaliana* (wild‐type Col‐0, and mutants *dcl234*, and *ago4*) seedlings were grown in a mix of soil, perlite, and vermiculite at a ratio of 1:1:1.

The plant growing conditions were a temperature of 22 ± 2°C and 60% relative humidity, with a 16‐h photoperiod (∼100 mmol m^−2^ s^−1^).

**Core Ideas**

- Phylogenetic analysis reveals miR9560 emerged in Rosanae and is conserved in Brassicaceae.
- Experimental proof shows that miR9560 is induced by cadmium (Cd) stress in a tissue‐specific pattern.
- miR9560 relies on the RNA‐directed DNA methylation pathway to regulate *BrpHMA2* under Cd stress.
- DNA methylation modulates *BrpHMA2* expression, enhancing Cd stress tolerance.

### 2.2 Phylogenetic analysis

Sequences of pri‐miR9560 were retrieved from miRBase (http://www.mirbase.org/) and PHYTOZOME v.13 (https://phytozome‐next.jgi.doe.gov). *F‐box* gene sequences were retrieved from PHYTOZOME v. 13 (https://phytozome‐next.jgi.doe.gov). Molecular phylogenetic analyses were performed in MEGA 5.0 using default values and the maximum likelihood method based on the Tamura‐Nei model (Tamura et al., 2011). The resulting tree was inferred from 1000 bootstrap replications, and the corresponding bootstrap values are presented in the trees with the posterior probabilities. The gene structure was supported by PHYTOZOME v. 13 (https://phytozome‐next.jgi.doe.gov), and the protein domain analysis was supported by SMART (http://smart.embl.de/) and NCBI (https://www.ncbi.nlm.nih.gov). TBTools was used to construct the consensus sequence of miR9560 in plants.

### 2.3 Secondary structure prediction

The predicted secondary structures of single‐stranded pri‐miRNA sequences were generated using the RNAfold web server (http://rna.tbi.univie.ac.at/cgi‐bin/RNAWebSuite/RNAfold.cgi).

### 2.4 RT‐qPCR

The leaves and roots of seedlings of *B. parachinensis* were used for real‐time quantitative reverse transcription polymerase chain reaction (RT‐qPCR). Total RNA was extracted from the samples using the RNAiso Plus reagent (TaKaRa) according to the manufacturer's protocol. RT‐qPCR was performed using TransStart Tip Green qPCR SuperMix (Transgen) on a Bio‐Rad CFX96 Real‐Time System (Bio‐Rad). *ACTIN*7 was used as the internal control gene for *B. parachinensis*. The primers used in this study are listed in Table S1.

### 2.5 RNA gel blotting

RNA gel blotting analysis for miRNA was performed as described previously (Park et al., 2002). Briefly, 10 µg of total RNA was separated on a polyacrylamide‐urea gel, transferred to a nylon membrane, and then analyzed using a biotin‐labeled oligonucleotide probe. The signals were measured using a ChemiScope chemical luminescence imaging system (CLiNX). U6 was employed as the control.

### 2.6 Transient gene expression assay in protoplasts

The pri‐miR9560 and the 1500 bp promoter of *BrpHMA2* were cloned from the DNA of *B. parachinensis* by PCR using PrimerStar (Takara). A 300 bp fragment containing the target site of miR9560 was cloned from the DNA of *B. parachinensis* fused upstream of the promoter of *BrpHMA2* and labeled as *pTBrpHMA2* (a constructed promoter containing a 300 bp fragment with the predicted miR9560 target site fused to the 1.5 kb promoter region of BrpHMA2). Next, the *pTBrpHMA2* fragment was fused upstream of the firefly luciferase gene (*LUC*) in the *pGreenII0800‐LUC* vector harboring *35S::RLuc* (Renilla luciferase, REN) (Hellens et al., 2005) to create a reporter construct. The precursor of miR9560 (*miR9560*) or the mutant of miR9560 (*miR9560 MUT*) was cloned into the *pGreen 0062SK* vector downstream of the 35S promoter to create an effector construct. Protoplast transformation and transient gene expression analysis were performed as described previously (S. Liu et al., 2022). About 10 µg of the reporter plasmid DNA and 40 µg of the effector were transformed per 1 × 10^7^ protoplasts of *B. parachinensis* or Arabidopsis leaves mediated by PEG4000/CaCl_2_. Transfected protoplasts were incubated in the dark for 16 h and then collected by centrifugation at 100 × g for 3 min at 4°C for further analysis. Dual LUC assays were performed using a Dual‐luciferase Reporter Assay Kit (Transgen). The promoter activity of *BrpHMA2* was indicated by the ratio of LUC to REN activity.

For the DNA methylation analysis, protoplast transfection was performed using about 50 µg of the effector plasmids per 1 × 10^7^ protoplasts.

### 2.7 DNA methylation analysis

Genomic DNA from the transformed protoplasts of *B. parachinensis* was incubated with the methylation‐specific endonuclease enzyme McrBC (New England Biolabs, USA) in a 100 µL total volume mixture containing genomic DNA, McrBC, reaction buffer, bovine serum albumin (BSA), and 1 mM GTP or ddH_2_O (as a negative control) at 37°C for over 16 h and then heated at 65°C for 20 min. The digested DNA was used for semi‐quantitative PCR analysis.

### 2.8 Statistical analysis

All data comprised at least three biological replicates. All data were analyzed by using the IBM SPSS Statistics 22 software, and statistically significant differences between samples were determined by Student's *t*‐test.

## 3 RESULTS

### 3.1 MiR9560 emerges in the Rosanae superorder

Our previous investigation found the presence of miR9560 in Cd‐induced miRNAs of *B. parachinensis* (Y. Liu et al., 2020). This is a novel 24‐nt lmiRNA. Upon querying the PmiREN (Plant MicroRNA Encyclopedia) database, we discovered that miR9560s were present in *Brassica rapa* (*B. rapa*)*, Citrus clementina* (*C. clementina*), *Citrus maxima* (*C. maxima*), and *Citrus sinensis* (*C. sinensis*). *B. rapa* and *B. parachinensis* are both members of the Brassicaceae family, whereas *C. clementina, C. maxima*, and *C. sinensis* belong to the Rutaceae family. The precursor sequences of miR9560 in the four plants were further collected from PmiREN. The miR9560 precursor sequences differed significantly between *B. rapa* and *C. clementina*, while the precursor sequences of miR9560a and miR9560b in *B. rapa* were highly conserved (Figure S1). A whole‐genome blast was done utilizing the precursors of *Bra‐miR9560a* and *Ccl‐miR9560* in Phytozome V13 to determine how *miR9560* evolved in the plant kingdom. The expected threshold value was set to three. All predicted secondary structures from the retrieved sequences were generated using the RNAfold web server (Figure S2). The 500 bp sequences featuring a hairpin structure from diverse plants were selected for the phylogenetic study. The molecular phylogenetic analysis reveals that a total of 45 miR9560 precursors are identified in 44 species, spanning 11 families (Brassicaceae, Fagaceae, Rosaceae, Malvaceae, Anacardiaceae, Salicaceae, Cucurbitaceae, Myrtaceae, Juglandaceae, and Rutaceae) belonging to the superorder Rosanae (Figure 1a). Among the 44 species, 25 belong to Brassicaceae and four belong to Fabaceae; there are three each in the Rosaceae and Rutaceae, two each in Malvaceae, Salicaceae, and Myrtaceae, and one each in the Fagaceae, Anacardiaceae, Cucurbitaceae, and Juglandaceae families. MiR9560 emerges in the Rosanae superorder and is widespread in the Brassicaceae family. Orthologs of the miR9560 precursor in most species are present as a single copy, whereas in *B. rapa* two copies are identified (Figure 1a). Thus, a duplication event of miR9560 likely occurred in *B. rapa* during evolution.

FIGURE 1Phylogenetic analysis of miR9560 related sequences. (a) Alignment of miR9560 precursor sequences from 44 species was performed using MAMG 5.0. The 500 bp nucleotide sequences encompassing the mature miRNA were extracted and aligned. The maximum likelihood method was employed to infer the consensus tree using 1000 bootstrap replicates (left). Each genus that the related species belongs to is denoted with a distinct color (middle). The positions of miR9560 and its neighboring gene structures within the species are illustrated on the right. (b) and (c) Comparison of miR9560 sequences between Brassicaceae and Rutaceae plants. (d) Secondary structure of pri‐miR9560 in Brassica rapa FPsc (B. rapa FPsc) and *Citrus clementina* (*C. clementina*).

**Figure:**
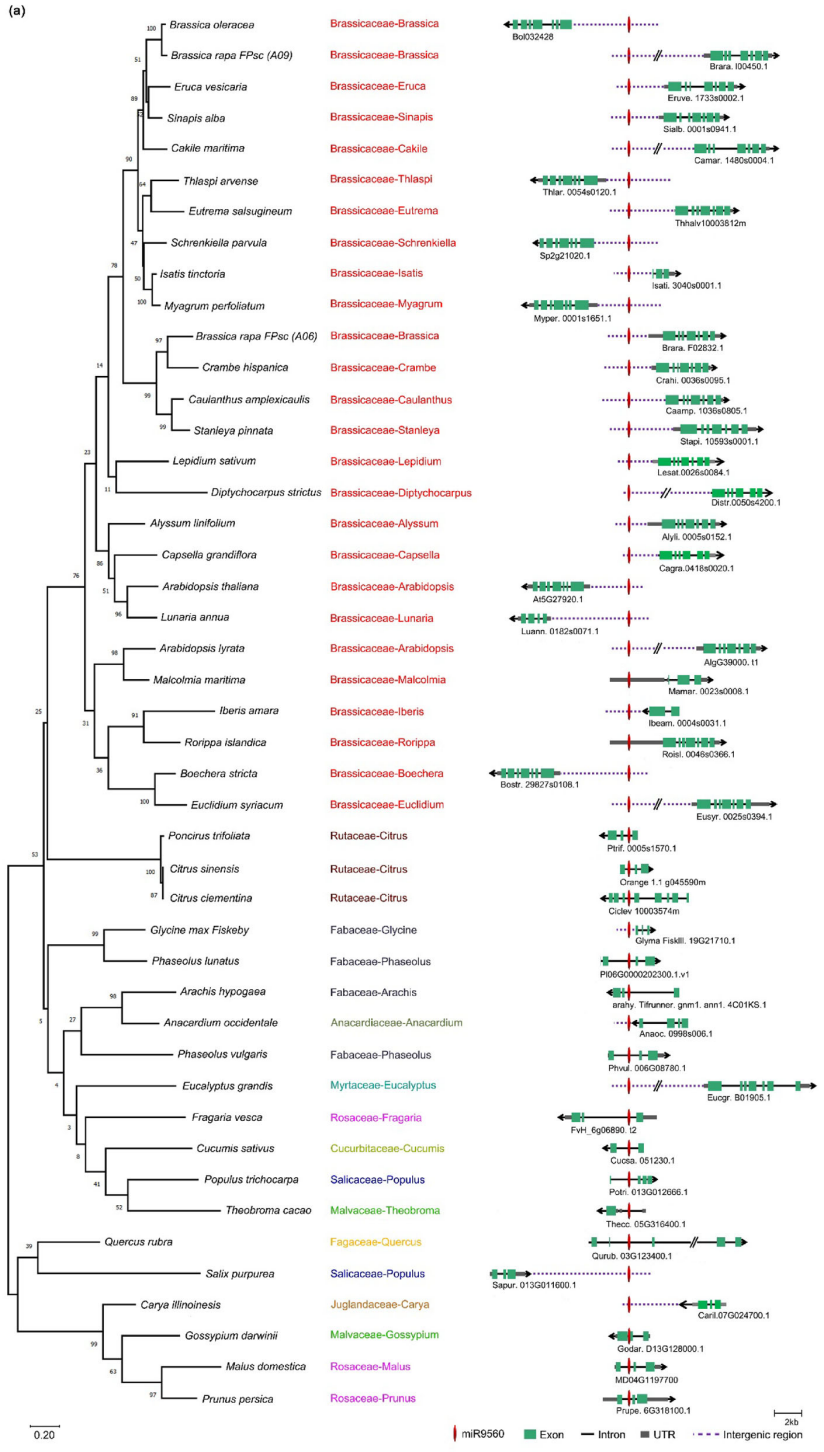

**Figure:**
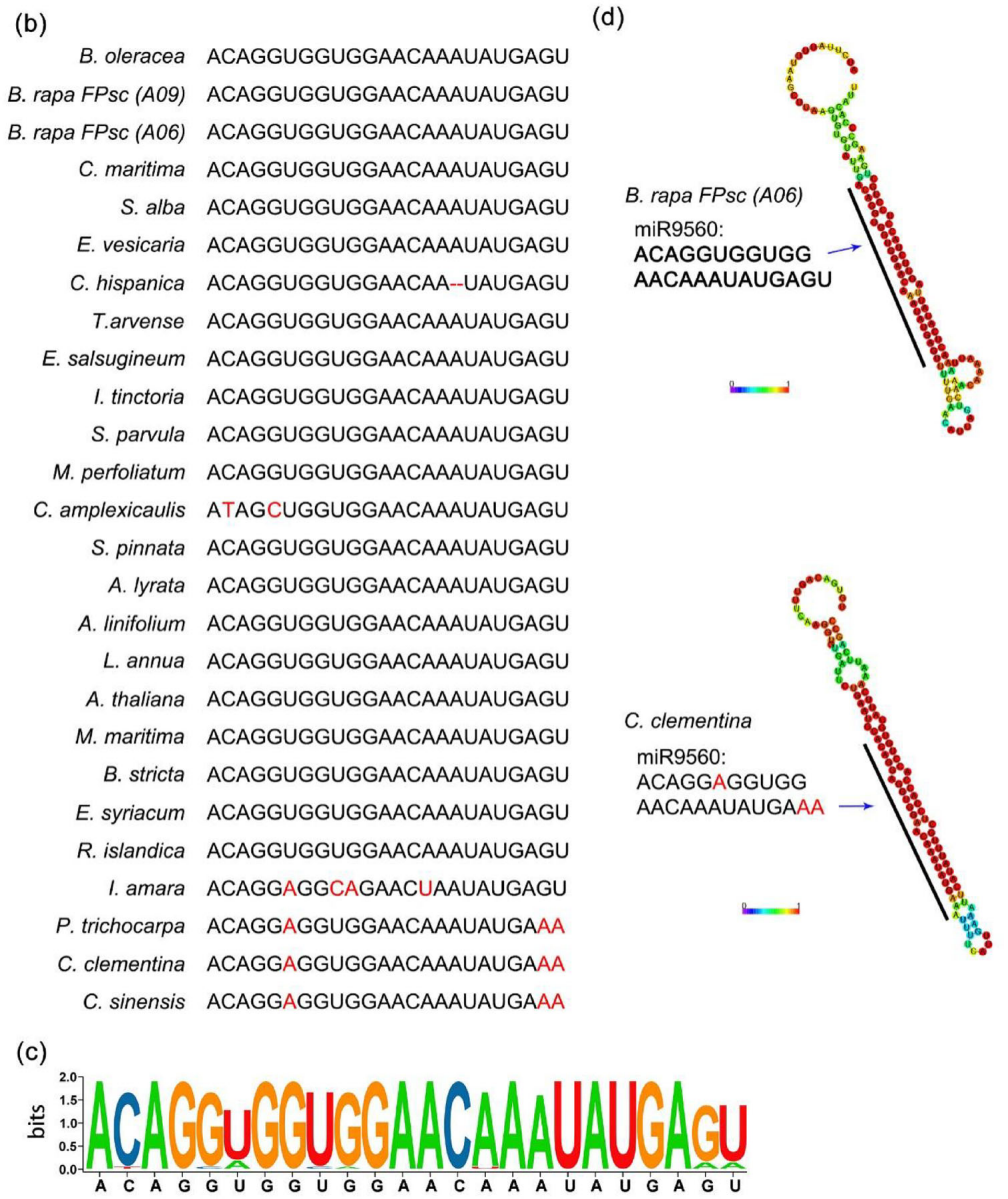

### 3.2 Genomic distribution of miR9560 across plant species

Genomic locations of miR9560 in the 44 species were further analyzed to investigate their conserved status. Statistical analysis shows that 28 members of the predicted *miR9560* gene (62.2%) are located in the intergenic region, and 17 members (37.8%) are in the intronic region (Figure S3). Among the 28 members within the intergenic region, 24 (85.7%) belong to Brassicaceae, with the remaining four belonging to Anacardiaceae, Myrtaceae, Salicaceae, and Juglandaceae.

Of the intronic group, 15 (88.2%) are within introns and belonged to Fabaceae, Rosaceae, Malvaceae, Rutaceae, Cucurbitaceae, Salicaceae, and Fagaceae, while two (11.8%) are in the 5′ UTR, both belonged to Brassicaceae (Figure S3). Among the 25 Brassicaceae species, 23 had the *miR9560* gene in the intergenic region. MiR9560 was previously identified through small RNA seq in *B. parachinensis* (Y. Liu et al., 2020), *C. clementina* (Taylor et al., 2017), and *B. rapa FPsc* (Zhang et al., 2018). Sequences alignment and logos analysis of miR9560 from Brassicaceae and Rutaceae revealed sequence variation at the sixth base, forming an asymmetric bulge (Figure 1b–d). This highlights that miR9560 has a wide distribution across plant families and specific sequence variations, particularly between Brassicaceae and Rutaceae.

A further analysis was conducted on the genes adjacent to *MIR9560* in various species. Phylogenetic analysis reveals that the neighboring or intragenic genes of *MIR9560* in the 44 species belong to the *F‐box* gene family (Figure S4). These genes encode proteins that contain an identical F‐box domain and can be categorized into subfamilies, such as LRR‐FBD, FBA1, FBA3, Kelch repeats, and domain unknown function (Xia et al., 2015). The domain analyses showed that these genes could be divided into two groups: one group encoded proteins belonging to the LRR‐FBD subfamily, including those in Brassicaceae species; the other group encoded proteins belonging to the PRR2 proteins containing an F‐box domain, including those in Rosaceae, Malvaceae, Fabaceae, Anacardiaceae, Salicaceae, and Cucurbitaceae (Figure S4). These findings indicate a frequent association between *miR9560* and *F‐box* genes.

### 3.3 Potential miR9560 target sites are adjacent to *HMA* genes

The target sites of miR9560 in various plant species were investigated to evaluate its putative function. The miR9560 sequence was used to perform BLAST searched against the whole genome of all 44 species using Phytozome V13. Potential target loci of miR9560 were identified in 28 species. Subsequently, 200 bp fragments encompassing the predicted target site of miR9560 were employed for molecular phylogenetic analysis using MEGA 5.0. The results showed that miR9560 target loci were present upstream of functional genes in 23 species of the Brassicaceae family, three species of Rutaceae, and two species of Myrtaceae (Figure 2a). Notably, all 28 of these functional genes belonged to the heavy metal transport ATPase (*HMA*) gene family.

FIGURE 2The potential miR9560‐targeted loci across different species. (a) Multiple 200 bp nucleotide sequences encompassing the possible miR9560‐targeted sites in 28 species were aligned using MAMG 5.0. The bootstrap consensus tree with values from 1000 bootstrap replicates (left) was constructed using the maximum likelihood method. Individual colors are employed to distinguish each group (middle). The potential miR9560 target sites and their adjacent gene structures in various species are depicted (right). (b) Phylogenetic analysis of the neighboring genes associated with miR9560‐targeted loci.

**Figure:**
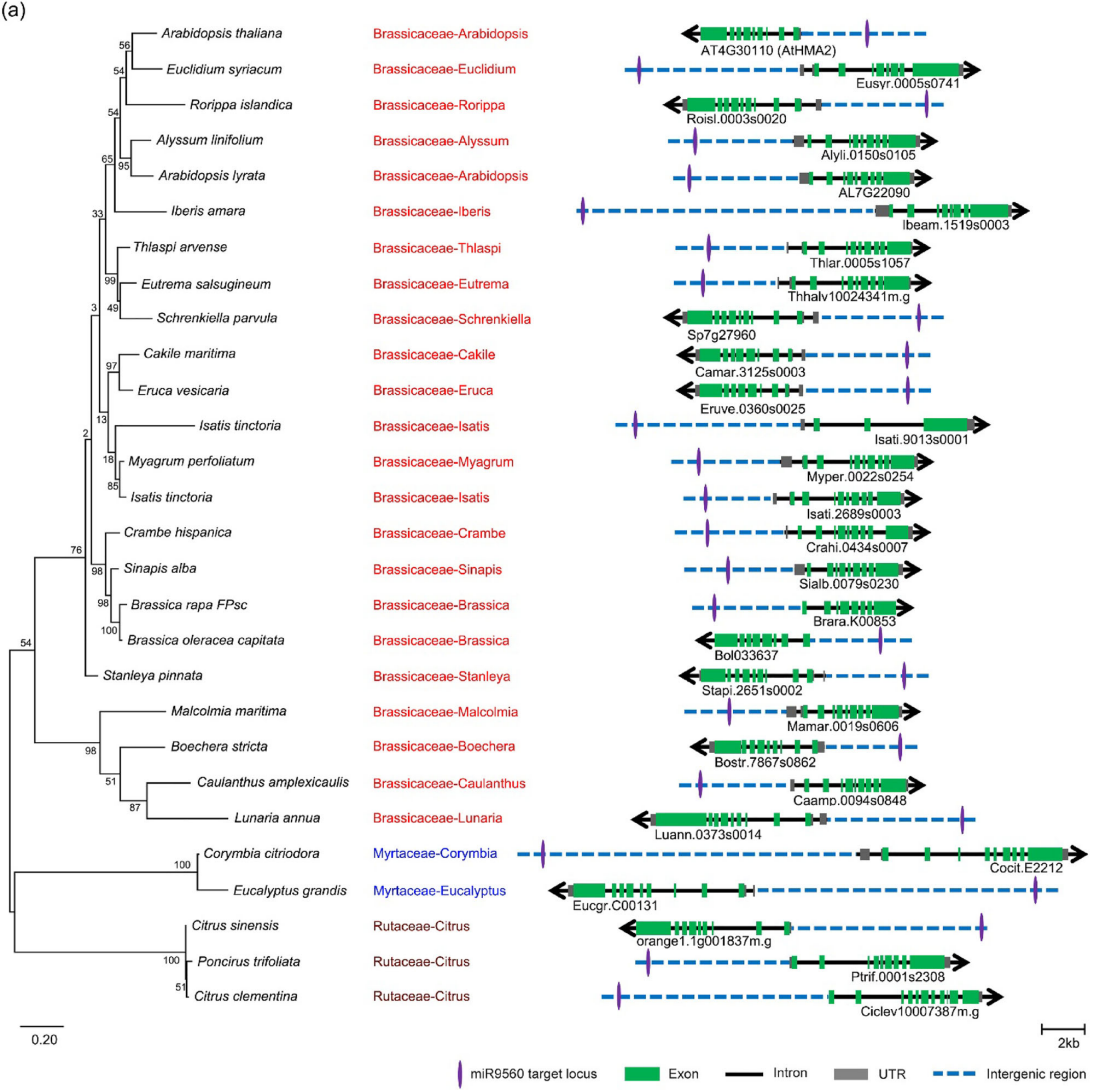

**Figure:**
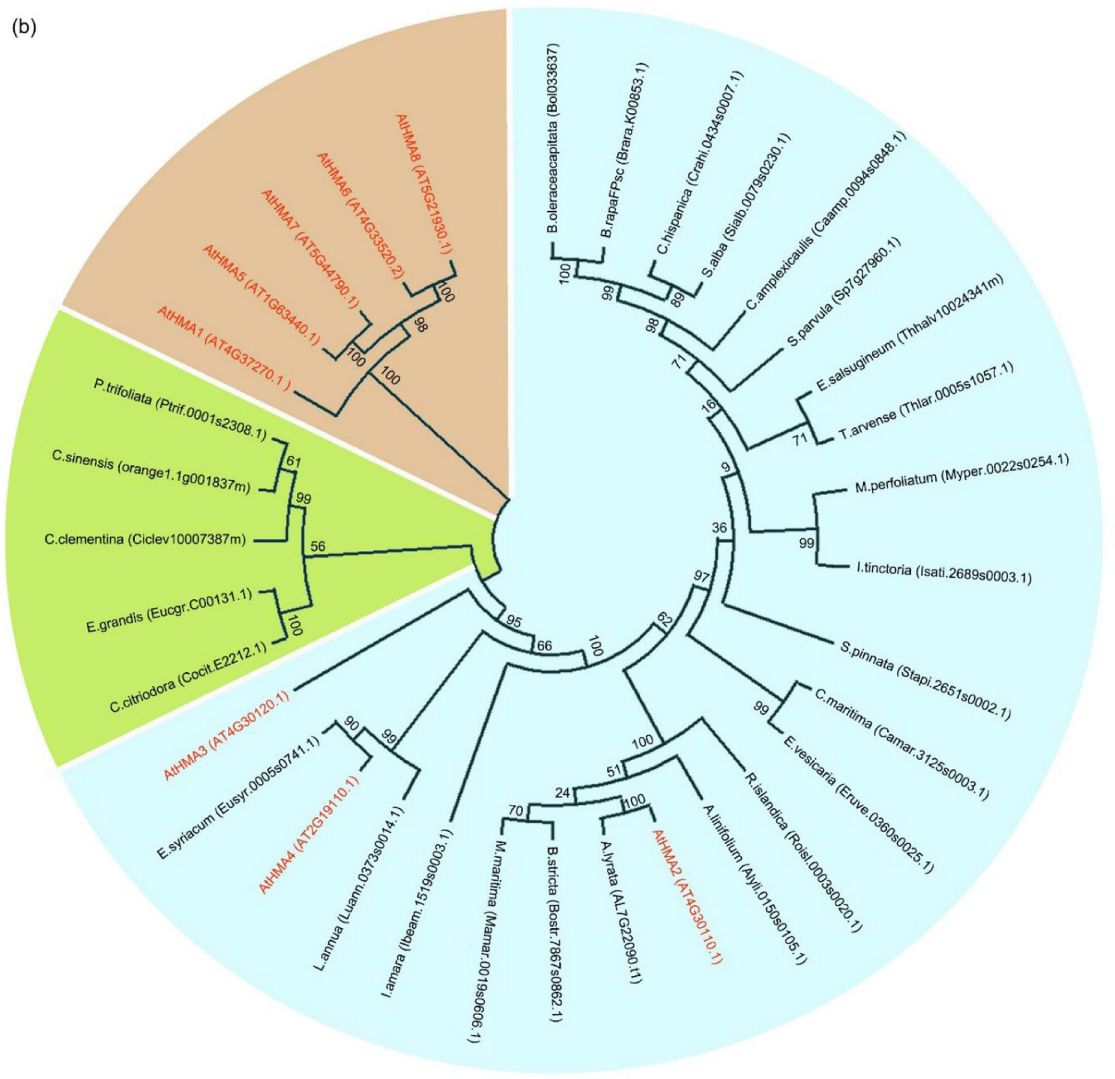

Molecular phylogenetic analyses of these genes in conjunction with the *HMA* family genes in Arabidopsis indicated that they may be grouped into three clusters: the first cluster contains genes from Brassicaceae along with *AtHMA2*, *AtHMA4*, and *AtHMA3*, suggesting that the functional genes adjacent to miR9560 target loci in Brassicaceae plants are most likely homologous to *AtHMA2*, *AtHMA4*, and *AtHMA3*. The second cluster contains the genes from Rutaceae and Myrtaceae, while the third comprises *AtHMA1* and *AtHMA5‐8* genes (Figure 2b). These findings characterized a potential miR9560‐*HMA* regulatory pathway prevalent in Brassicaceae, Rutaceae, and Myrtaceae.

### 3.4 Differential expression of miR9560 in *B. parachinensis* under Cd stress

**FIGURE 3:**
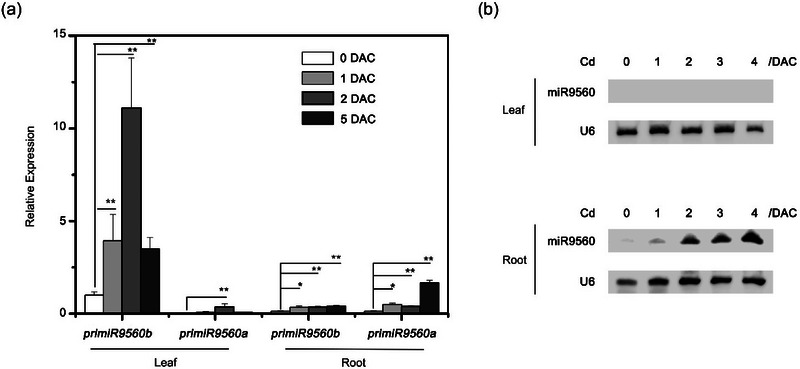
Induction of miR9560 expression by Cd stress in *Brassica parachinensis*. (a) Relative expression of the two miR9560 precursors in *Brassica parachinensis*. Twelve‐day‐old hydroponic seedlings of *B. parachinensis* at 0–5 days after 50 µM Cd(NO_3_)_2_ treatment (DAC) were harvested. The expression of two miR9560 transcripts (pri‐miR9560a, pri‐miR9560b) was analyzed by real‐time quantitative reverse transcription polymerase chain reaction (RT‐qPCR). (b) RNA gel blotting analysis of miR9560 in leaves and roots of *B. parachinensis*. Asterisks indicate significant differences with respect to means of the control plants (**^∗^** *p* < 0.05, **^∗∗^** *p* < 0.01; Student's *t*‐test).

To explore how Cd induces miR9560 expression, two genes in *B. parachinensis*, namely, *MIR9560a* and *MIR9560b*, were predicted by PmiREN to produce mature miRNAs with identical sequences. The levels of pri‐miR9560 in seedlings subjected to Cd stress (Figure S5) were analyzed by RT‐qPCR. Results showed that both pri‐miR9560a and pri‐miR9560b expressions were induced by Cd stress in both leaves and roots. However, the increase in pri‐miR9560a expression was lower compared to pri‐miR9560b, with the latter showing particularly high levels in the leaves after Cd exposure (Figure 3a). RNA gel blotting demonstrated the accumulation of mature miR9560 in the roots of *B. parachinensis* after Cd exposure (Figure 3b). Surprisingly, no blot signals were detected in the leaf samples despite the high expression level of pri‐miR9560b in the Cd‐exposed leaves (Figure 3a). These results indicate an inconsistency between the expression patterns of pri‐miR9560 and miR9560.

### 3.5 Conservation of Cd‐inducible miR9560 expression across Brassica species

**FIGURE 4:**
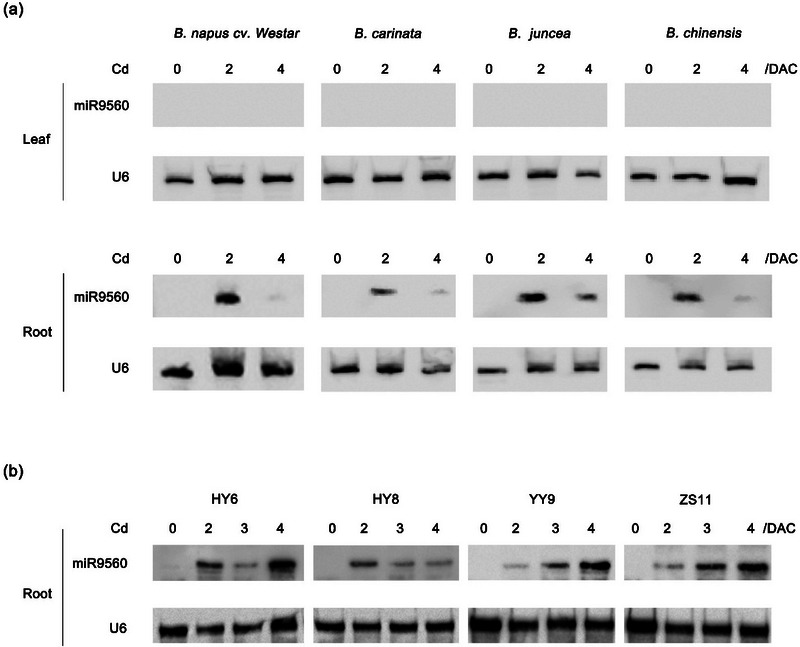
Induction of miR9560 expression by Cd stress across several species in the *Brassica* genus. (a) RNA gel blotting analysis of miR9560 in leaves and roots of several *Brassica* species. (b) RNA gel blotting analysis of miR9560 in roots of several cultivars in *Brassica napus*. Twelve‐day‐old hydroponic seedlings at 0–4 days after 50 µM Cd(NO_3_)_2_ treatment (DAC) were used for the assay. U6 served as the loading control.

*Brassica* is one of the most important genera in the Brassicaceae family, encompassing numerous popular vegetable and oil crops. According to the U's Triangle (Song et al., 2021), the genus includes three diploid species (*B. rapa*, AA, *n* = 10; *B. nigra*, BB, *n* = 8; and *B. oleracea*, CC, *n* = 9) and three amphidiploid allotetraploid species (*B. napus*, AACC, *n* = 19; *B. juncea*, AABB, *n* = 18; and *B. carinata*, BBCC, *n* = 17), which were crossed, achieving chromosome doubling from the diploids. To investigate the response of miR9560 to Cd in *Brassica* species, hydroponically grown seedlings of *B. napus* ‘Westar’ (AACC), *B. carinata* (BBCC), *B. juncea* (AABB), and *B. chinensis* (AA) were exposed to Cd stress (Figure S5), and their leaves and roots were harvested for RNA gel blotting analysis. The results demonstrated that Cd stress substantially upregulated the expression of miR9560 in the roots of all four tested species (Figure 4a), mirroring the expression pattern observed in *B. parachinensis*. Further experiments were carried out to assess the impact of Cd‐induced stress on various oilseed rape cultivars prevalently cultivated in different regions in China, including ZS11 in the Yangtze River valley and HY6, HY8, and YY9 in the mountainous area. RNA gel blotting analysis revealed that miR9560 could be induced by Cd stress in the roots of all tested varieties (Figure 4b). These findings suggested that the Cd‐inducible expression pattern and potential function of miR9560 may be conserved across *Brassica* species.

### 3.6 MiR9560 inhibits the transcription of *BrpHMA2* in *B. parachinensis*

**FIGURE 5:**
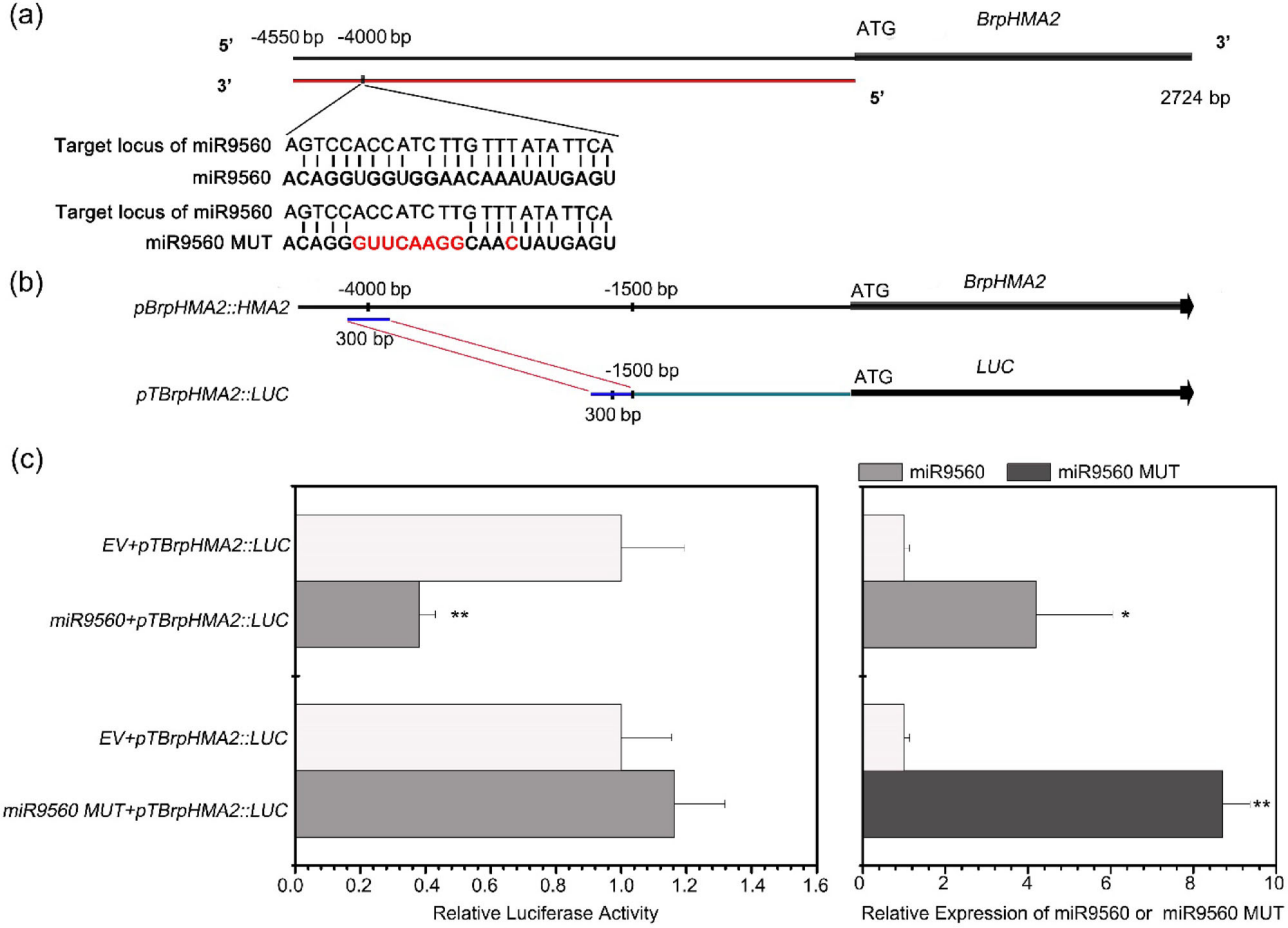
Suppression of *BrpHMA2* transcription by miR9560. (a) Sequences of miR9560 and its mutant, cloned into *pGreen 0062SK* serving as the effectors. (b) Reporter constructs *pTBrpHMA2::LUC* containing the predicted target region and the *BrpHMA2* promoter. (c) Impact of miR9560 on *BrpHMA2* transcription. Relative luciferase activity was assayed in *Brassica parachinensis* protoplasts (left) transformed with *pTBrpHMA2::LUC* and either *miR9560*, *miR9560 MUT*, or the empty vector of *pGreen 0062SK* (empty vector, *EV*). RT‐qPCR was performed simultaneously to analyze the expression of *miR9560* or *miR9560 MUT* in the *B. parachinensis* protoplasts (Right). Asterisks indicate significant differences with respect to the means of the control samples (**^∗^** *p* < 0.05, **^∗∗^** *p* < 0.01; Student's *t*‐test).

To investigate the role of miR9560 in *B. parachinensis*, the target site was predicted using psRNATarget. A specific site was identified upstream of the *BrpHMA2* gene, responsible for Cd transport in an afflux manner (S. Liu et al., 2022) (Figure 5a). To determine whether miR9560 could regulate the transcription of *BrpHMA2*, a protoplast transient expression system was utilized. The pri‐miR9560 and a mutant version of pri‐miR9560 (Figure 5a) controlled by the 35S promoter (*miR9560*, *miR9560 MUT*, respectively) were constructed as the effectors. A 300 bp fragment containing the predicted miR9560 target site was fused to the 1.5 Kb promoter region of *BrpHMA2* (Figure 5b). The recombinant promoter (named *pTBrpHMA2*) was employed to drive the reporter gene *LUC* (*pTBrpHMA2::LUC*) for expression as a reporter (Figure 5b). The effectors and the empty vector (*EV*) *pGreen 0062SK* (as the control) were co‐transformed with the reporter into *B. parachinensis* leaf protoplasts. The results showed that overexpression of miR9560 significantly repressed the expression of *LUC*, whereas the mutated miR9560 had no effect (Figure 5c). These findings suggest that miR9560 inhibit the transcription of *BrpHMA2*.

### 3.7 MiR9560 enhances DNA methylation of the upstream region of the *BrpHMA2* gene via the RdDM pathway

**FIGURE 6:**
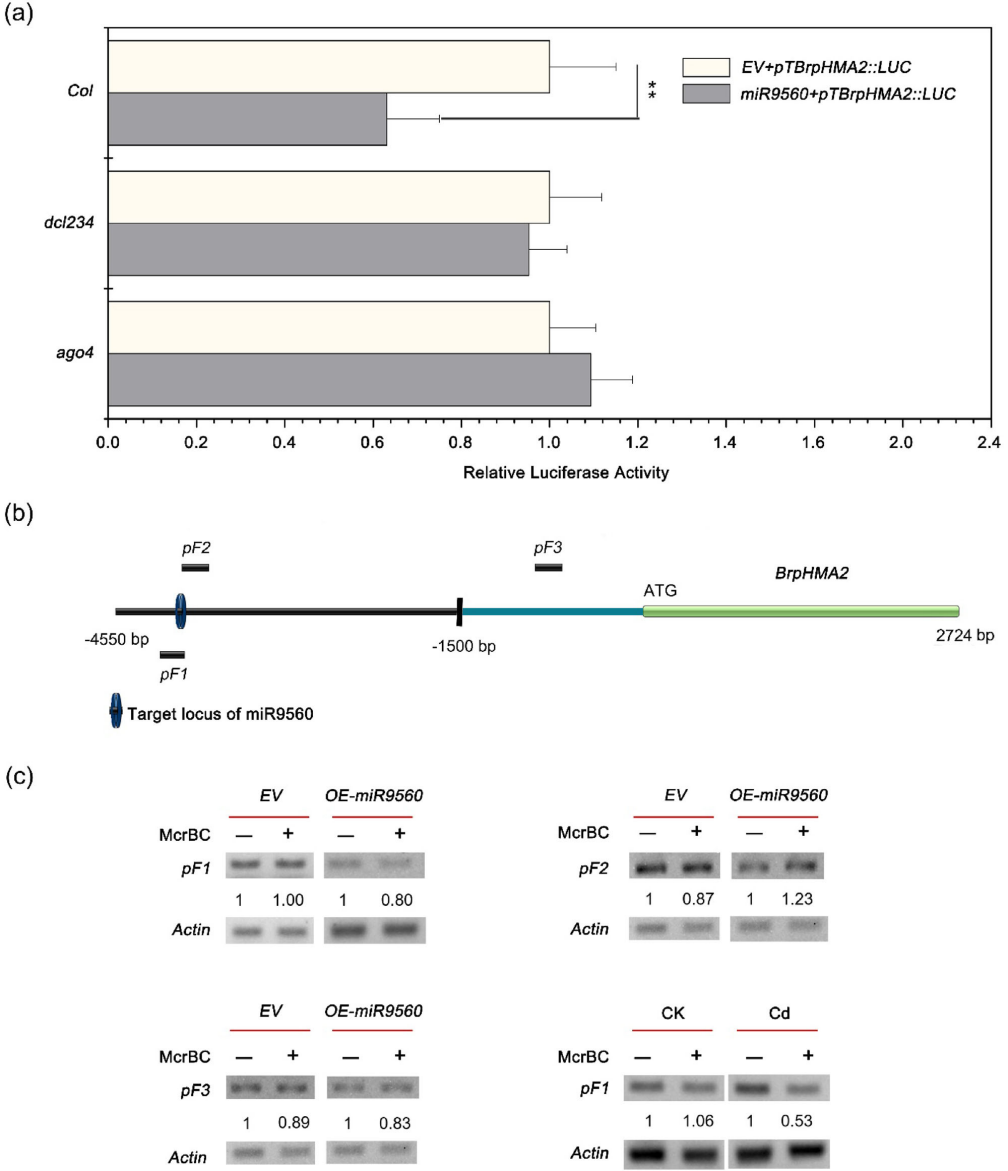
Increase in DNA methylation of *BrpHMA2*’s upstream region via the RdDM pathway induced by miR9560. (a) MiR9560 did not reduce the transcription of *BrpHMA2* in *dcl234* or *ago4* protoplasts. Relative luciferase activity was measured in Arabidopsis lines (Col‐0, *dcl234*, or *ago4*) using protoplasts transformed with *pTBrpHMA2::LUC* and *miR9560* or empty vector (*EV*). (b) Schematic diagram of the fragments containing predicted methylation sites (*pF1–pF3*) upstream of *BrpHMA2*. (c) The methylation levels in the upstream region of *BrpHMA2* were affected by either miR9560 or exposure to Cd‐induced stress. Protoplasts of *Brassica parachinensis* transformed with different vectors (*EV*, *miR9560*), or the *B. parachinensis* seedlings treated with or without Cd (Cd, CK, the right bottom) were harvested for the DNA methylation analysis. The numbers represent the relative abundance of blots. Asterisks indicate significant differences for the means of the control samples (**^∗∗^** *p* < 0.01; Student's *t*‐test).

Previous studies have reported miRNA‐directed DNA methylation near both the miRNA locus and its target loci (Wu et al., 2010). We postulated that miR9560 repressed the expression of *BrpHMA2* via the RdDM pathway. Consequently, miR9560 should be potentially generated by DCL3 and subsequently incorporated into AGO4 to regulate DNA methylation (Chen & Rechavi, 2022; Wu et al., 2010). To validate this hypothesis, an Arabidopsis mutant with a defect in 24‐nt sRNA synthesis (*dcl234*) and the mutant with an impaired RdDM pathway (*ago4*) were used for analyzing the function of miR9560. The plasmid *pTBrpHMA2::LUC* was co‐transformed with *miR9560* or *EV* into protoplasts derived from Arabidopsis lines (Col‐0, *dcl234*, or *ago4*). The results showed that the repression of *BrpHMA2* by miR9560 was detected only in the Col‐0 protoplasts but not in the mutants *dcl234* or *ago4* (Figure 6a), suggesting that miR9560 mediated transcriptional regulation of *BrpHMA2* is dependent on the RdDM pathway.

DNA methylation levels of the regions upstream of *BrpHMA2* were analyzed using McrBC semi‐quantitative PCR. Methylation was detected at the predicted sites close to the miR9560 target locus (pF1, pF2) and another site in the promoter of *BrpHMA2* (pF3) (Figure 6b). Increased methylation levels at pF1 were observed in both in the *B. parachinensis* protoplasts with miR9560 overexpression and in the plants under Cd stress, while higher methylation level at pF3 was detected upon miR9560 overexpression (Figure 6c). These findings suggest that miR9560 may repress the transcription of *BrpHMA2* by enhancing the DNA methylation level in its upstream region under Cd stress.

## 4 DISCUSSION

### 4.1 Evolutionary conservation and species‐specific expression of MiR9560 in the Rosaanae superorder

The criteria for plant miRNA annotation as proposed by Axtell and Meyers (2018) and Meyers et al. (2008) emphasize the hairpin structure of miRNA precursors as the primary feature for identifying plant miRNAs. Following these criteria, miR9560 was confirmed as a 24‐nt miRNA in *B. parachinensis* (Y. Liu et al., 2020). Thus far, in addition it was detected in sRNA‐seq of *B. parachinensis* seedlings after exposure to Cd stress (Y. Liu et al., 2020); miR9560 has been exclusively identified in Rutaceae family (Figure S6). It was initially detected in the emerging phase of polyembryonic citrus, where it exhibited high expression levels in nuclear embryo initiation cells (Long et al., 2016). Despite these early findings, further research into miR9560 has been limited. In this study, we report its presence in the roots of *B. parachinensis* and several *B. napus* cultivars after Cd stress (Figures 3 and 4). Whole‐genome blast by the miR9560 precursor sequences with a hairpin structure showed that they emerged in 44 species, among them 25 belonged to Brassicaceae (Figure 1a). The plant species harboring miR9560 sequences and target sites were mapped on the phylogenetic tree created by H. T. Li et al. (2021) (Figure S6). The results clearly show that the hairpin structure of miR9560 emerges in the superorder Rosanae, encompassing the families Brassicaceae, Rutaceae, Rosaceae, Fagaceae, etc. (Figure S6), and it is broadly conserved across the Brassicaceae (Figure 1a). This distribution pattern suggests a potential evolutionary conservation of miR9560 among these species.

The miR9560 precursor sequences were also identified in the *A. thaliana* genome; our RNA gel blotting experiments failed to detect any miR9560 signals in Arabidopsis, both with or without Cd stress (data not shown). Additionally, miR9560 was only identified in a single sRNA‐seq dataset of the Arabidopsis silique, with a very low occurrence (Table S2). Thus, the transcription regulation and biogenesis of miR9560 remain unclear.

This study highlights the prevalence of miR9560 precursor hairpin structure in the superorder Rosanae and its rare, species‐specific expression, pointing to the need for further investigation into its functional role.

### 4.2 Tissue‐specific expression and transport of miR9560 in Brassica under Cd stress

In our lab, miR9560 was initially detected in a previous sRNA‐seq analysis of Cd‐stressed *B. parachinensis* (Y. Liu et al., 2020). Here, we further confirmed the expression of miR9560 in the roots of all tested *Brassica* plants when exposed to Cd stress (Figures 3 and 4). This result indicates that miR9560 may be widely present in *Brassica* plants with similar Cd‐inducible expression and tissue‐specificity patterns.

Additionally, the expression levels of pri‐miR9560 and mature miR9560 exhibited different tissue‐specific patterns when *B. parachinesis* was subjected to Cd‐induced stress. Notably, mature miR9560 accumulated in roots, whereas pri‐miR9560b accumulated predominantly in the shoots. However, the induced transcripts of pri‐miR9560a and pri‐miR9560b were detected at very low levels in the roots (Figure 3). These results pose two intriguing questions: Is the pri‐miR9560 or its mature form, miR9560, transported over long distances from the shoot to the root, resulting in the accumulation of miR9560 in roots after Cd exposure? Does the producing or stability of miR9560 require specific factors that are exclusively present in roots, but absent in leaves?

The long‐distance migration of many miRNAs has been demonstrated. For example, upon grafting, miR399 migrated from shoots that lacked sufficient inorganic phosphorus (Pi) to roots that had an adequate supply of phosphorus (Lin et al., 2008). MiR2111 derived from shoots could promote nodule formation in the roots of *Lotus japonicus* (Okuma et al., 2020; Tsikou et al., 2018). To further analyze the tissue‐specific differential expression pattern of miR9560, grafting experiments with wild‐type plants and miR9560 mutants could be conducted to observe the mobility of this lmiRNA or its precursor. The stable transgenic system in *B. parachinensis* should facilitate addressing this question.

These findings expand our comprehension of how miR9560 is expressed, providing valuable insights into its possible roles in plant stress responses.

### 4.3 Potential target sites of miR9560

It has been reported that lmiRNAs can target DNA and regulate the transcription of gene adjacent to the target locus (Wu et al., 2010; Zheng et al., 2024). In this study, the predicted target loci of miR9560 were identified in the genome of 28 species out of the 44 species possessing the miR9560 precursor sequences. Notably, 23 of these species belong to the Brassicaceae family. All the predicted target sites are specifically located upstream of functional genes belonging to the *HMA* gene family (Figure 2).

In Brassicaceae, the functional genes adjacent to the miR9560 target loci are homologous to *AtHMA2*, *AtHMA4*, and *AtHMA3*. Protein HMA2, HMA4, and HMA3 serve as the transporters on membrane or tonoplast and play roles in heavy metal transport and homeostasis in plants (Hussain et al., 2004; Liu et al., 2017; Qiao et al., 2018; Satoh‐Nagasawa et al., 2012; Wong & Cobbett, 2009). This finding suggests a correlation between *miR9560* and *HMA* genes in species belonging to the superorder Rosanae that possess both miR9560 and its predicted target loci, particularly in the Brassicaceae family plants.

### 4.4 MiR9560 may modulate the transcription of *BrpHMA2* in *B. parachinensis*

It has been reported that lmiRNAs can direct cytosine DNA methylation at their producing locus as well as their target locus (Wu et al., 2010). However, this methylation is typically confined to a region within 80 nt surrounding the target site (Wu et al., 2010). For example, a rice‐specific 24‐nt lmiRNA, Osa‐miR1873, directs DNA methylation of the target sites at LOC_Os05g01790 loci by loading into AGO4 (Wu et al., 2010) and regulates rice blast disease resistance (Zhou et al., 2020). Similarly, Zheng et al. (2024) demonstrated that a 24‐nt miR2863c, associated with OsAGO2, targets and mediates methylation of the OsNAC300 promoter region, inhibits the transcription of OsNAC300, and regulates leaf senescence in rice (Zheng et al., 2024). In this study, we analyzed the methylation levels of three predicted methylation loci within a 4550 bp promoter region of *BrpHMA2* gene. The results showed an elevation in DNA methylation levels at two sites when miR9560 was overexpressed in a protoplast system: one located near the miR9560 target locus (F1) and another situated >2 kb away from the target locus (F3). Simultaneously, a decrease in the transcription level of the reporter gene, which was driven by the extended promoter, was observed (Figures 5 and 6). In addition, the DNA methylation level at the predicted methylation site (F1) was increased in the Cd‐stressed *B. parachinensis* plants (Figure 6). Combined with the heterologous expression and methylation analysis in Arabidopsis mutants *dcl234* and *ago4* (Figure 6a), the results indicate that miR9560 may downregulate *BrpHMA2* expression through the RdDM pathway. However, both *BrpHMA2* (S. Liu et al., 2022) and miR9560 (Figure 3) could be induced by Cd stress in *B. parachinensis*; thus, some other positive regulators may act to reverse the repression of *BrpHMA2* expression by miR9560. BrpNAC895 and BrpABI449, which belong to the NAC transcriptional factors and bZIP transcriptional factors families, respectively, have also been demonstrated to be co‐regulators in *BrpHMA2* transcription. BrpNAC895 serves as a transcription activator of *BrpHMA2*, while BrpABI449 represses *BrpHMA2* by binding to the *BrpHMA2* promoter or interacting with the BrpNAC895 protein (S. Liu et al., 2022). We postulate that miR9560 coordinates with the repressor BrpABI449 and the activator BrpNAC895 to fine‐tune the transcription of *BrpHMA2*.

HMA2 is a member of the heavy metal ATPase family. Previous studies have suggested that homologs of HMA2, such as AtHMA2, TaHMA2, and OsHMA2, are responsible for Zn^2+^/Cd^2+^ efflux from cells, being involved in Zn and Cd loading to the xylem and participating in the root‐to‐shoot translocation of Zn/Cd in different plants (Hussain et al., 2004; Qiao et al., 2018; Satoh‐Nagasawa et al., 2012; Takahashi et al., 2012; Tan et al., 2013). In our previous study, BrpHMA2 was identified as a plasma membrane‐localized afflux‐type Cd transporter involved in the Cd^2+^ uptake and long‐distance transport in plants (S. Liu et al., 2022). Both *BrpHMA2* and miR9560 are Cd‐inducible, and *BrpHMA2* expression is regulated by miR9560. This suggests that miR9560 is involved in Cd translocation in *B. parachinensis* by the RdDM pathway.

Even though the phylogenetic analysis shows that *MIR9560* genes in the genome are typically adjacent to the *F‐box* gene family (Figure S4), neither the target sites nor the potential methylation sites were predicted in both *MIR9560* gene and *F‐box* gene in *B. parachinensis*. The potential regulatory interactions between miR9560 and *F‐box* genes are still unknown.

Considering the findings for miR9560 target loci and the genes near these loci (Figure 2a), we concluded that the miR9560‐*HMAs* pathway is specifically present in some families in the superorder Rosanae and widely present in Brassicaceae family; therefore, the miR9560‐*HMAs* pathway is proposed to function conservatively through RdDM and to be involved in regulating the transport of heavy metal ions (i.e., Cd^2+^). Our findings once again illustrate the role of 24‐nt miRNA in mediating DNA methylation and participating in plant stress response. Further studies can be conducted on genetically modified *B. parachinensis* and give a detailed analysis of the downstream effects of miR9560‐*HMA2* in plant responses to Cd^2+^ stress. Additionally, exploring the potential role of miR9560 in other plant families and species could provide further insights into its evolutionary conservation and functional importance in plant biology.

## AUTHOR CONTRIBUTIONS

**Yongsheng Bai**: Formal analysis; methodology; validation; writing—original draft. **Xiaoting Wang**: Formal analysis; validation; visualization. **Shahid Ali**: Methodology; writing—original draft; writing—review and editing. **Yang Liu**: Validation; visualization. **Jiannan Zhou**: Resources. **Meiting Liu**: Investigation; validation. **Shuai Liu**: Conceptualization; formal analysis; methodology; validation; writing—original draft. **Yulin Tang**: Conceptualization; data curation; funding acquisition; investigation; methodology; project administration; resources; writing—original draft; writing—review and editing.

## CONFLICT OF INTEREST STATEMENT

The authors declare no conflicts of interest.

## Supporting information




**FIGURE S1** A comparison of the predicted pri‐miR9560 sequences in various plants
**FIGURE S2** The predicted secondary structure of pri‐miR9560 in plant species by using the RNAflod web server
**FIGURE S3** Locations of miR9560s scattered at the intergenic and intronic regions in different species. This is the statistical analysis of FIGURE 1a
**FIGURE S4** Phylogenetic analysis of the genes nearby miR9560 in plants
**FIGURE S5** *Brassica* plants treated with cadmium
**FIGURE S6** The existence of miR9560 in flowering plants
**Table S1** Primer sequences used for RNA gel blotting and PCR
**Table S2** miR9560 in *Arabidopsis thaliana*

## Data Availability

All data supporting the findings of this study are available within the Supporting Information. These files include detailed experimental data, raw and processed datasets, and additional information necessary to reproduce the results. For further inquiries, data requests, or access to specific datasets, readers are encouraged to contact the corresponding author. All authors have contributed to and approved the data availability statement, ensuring transparency and accessibility of the research data.
