# Supplementary material for: A 24‐nt miR9560 modulates the transporter gene BrpHMA2 expression in Brassica parachinensis
Source: Plant Genome. 2025 Mar 19;18(1):e70013. doi: 10.1002/tpg2.70013 (PMC11922684; doi:10.1002/tpg2.70013)
Supplement: Supplementary file 1 — FIGURE S1 A comparison of the predicted pri‐miR9560 sequences in various plants FIGURE S2 The predicted secondary structure of pri‐miR9560 in plant species by using the RNAflod web server FIGURE S3 Locations of miR9560s scattered at the intergenic and intronic regions in different species. This is the statistical analysis of FIGURE 1a FIGURE S4 Phylogenetic analysis of the genes nearby miR9560 in plants FIGURE S5 Brassica plants treated with cadmium FIGURE S6 The existence of miR9560 in flowering plants Table S1 Primer sequences used for RNA gel blotting and PCR Table S2 miR9560 in Arabidopsis thaliana [file TPG2-18-e70013-s001.docx]

**Supporting Information Available**

**FIGURE S1** A comparison of the predicted pri-miR9560 sequences in various plants

**FIGURE S2** The predicted secondary structure of pri-miR9560 in plant species by using the RNAflod web server

**FIGURE S3** Locations of miR9560s scattered at the intergenic and intronic regions in different species. This is the statistical analysis of FIGURE 1a

**FIGURE S4** Phylogenetic analysis of the genes nearby miR9560 in plants

**FIGURE S5** *Brassica* plants treated with cadmium

**FIGURE S6** The existence of miR9560 in flowering plants

**Table S1** Primer sequences used for RNA gel blotting and PCR

**Table S2** miR9560 in *Arabidopsis thaliana*


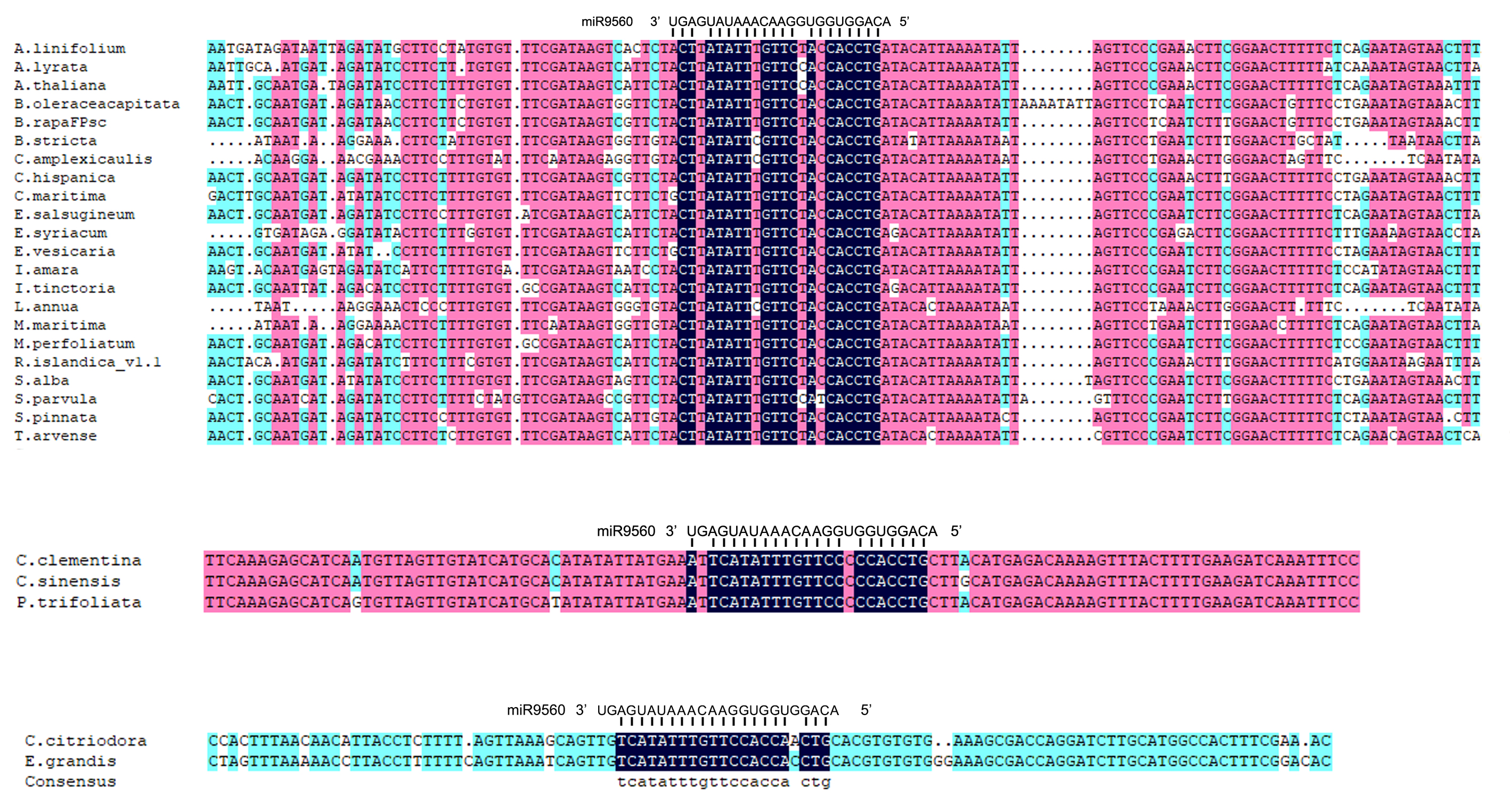


**FIGURE S1 A comparison of the predicted pri-miR9560 sequences in various plants**


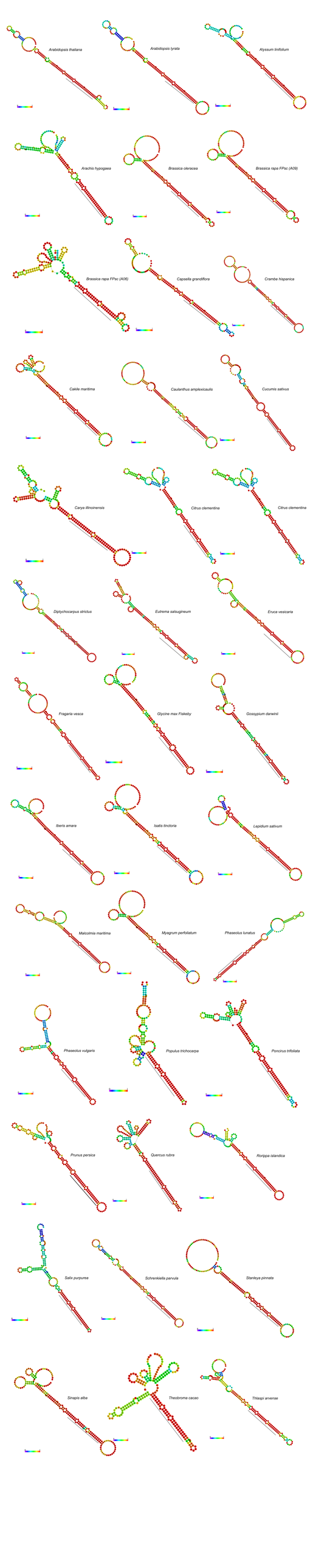


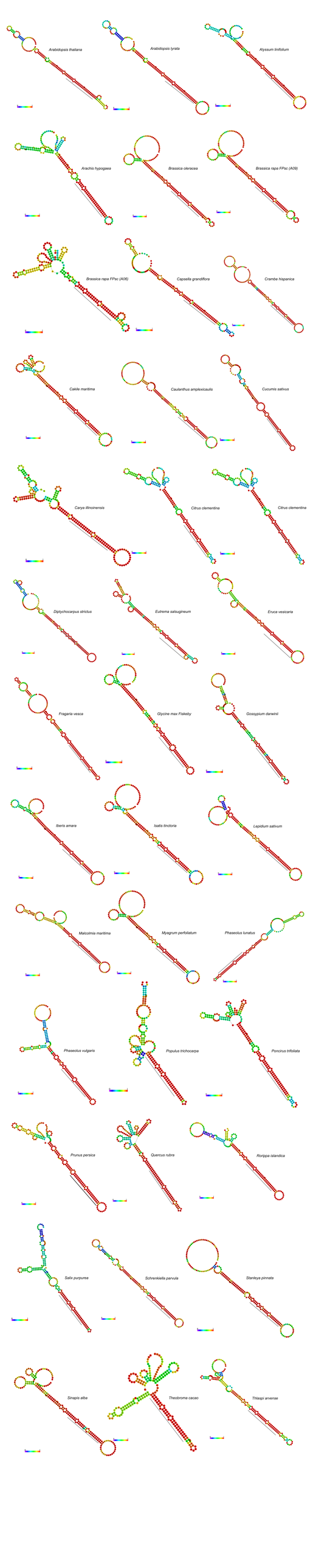


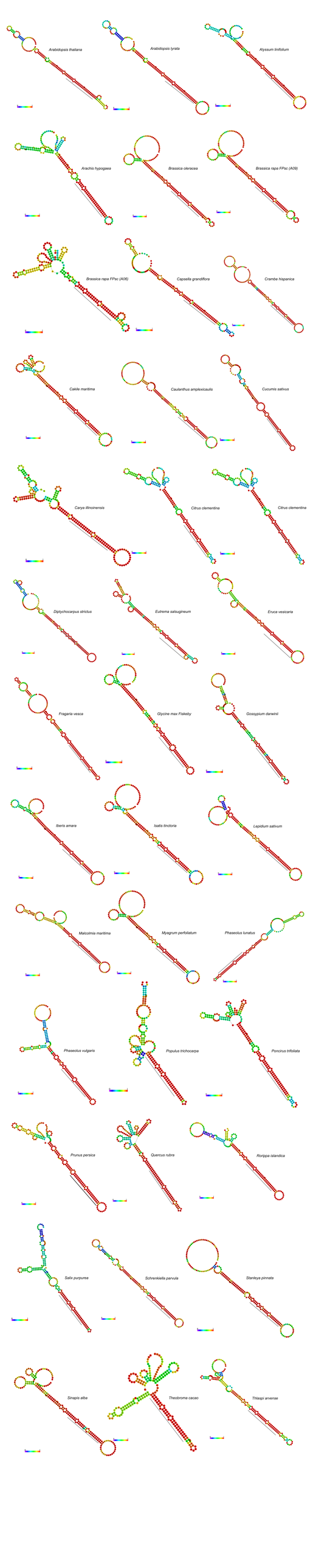


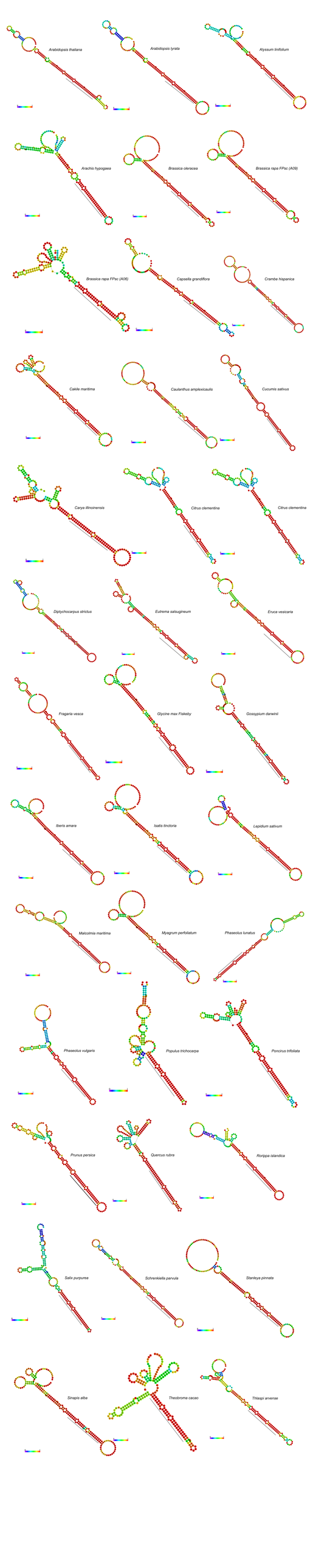


**FIGURE S2 The predicted secondary structure of pri-miR9560 in plant species by using the RNAflod web server**


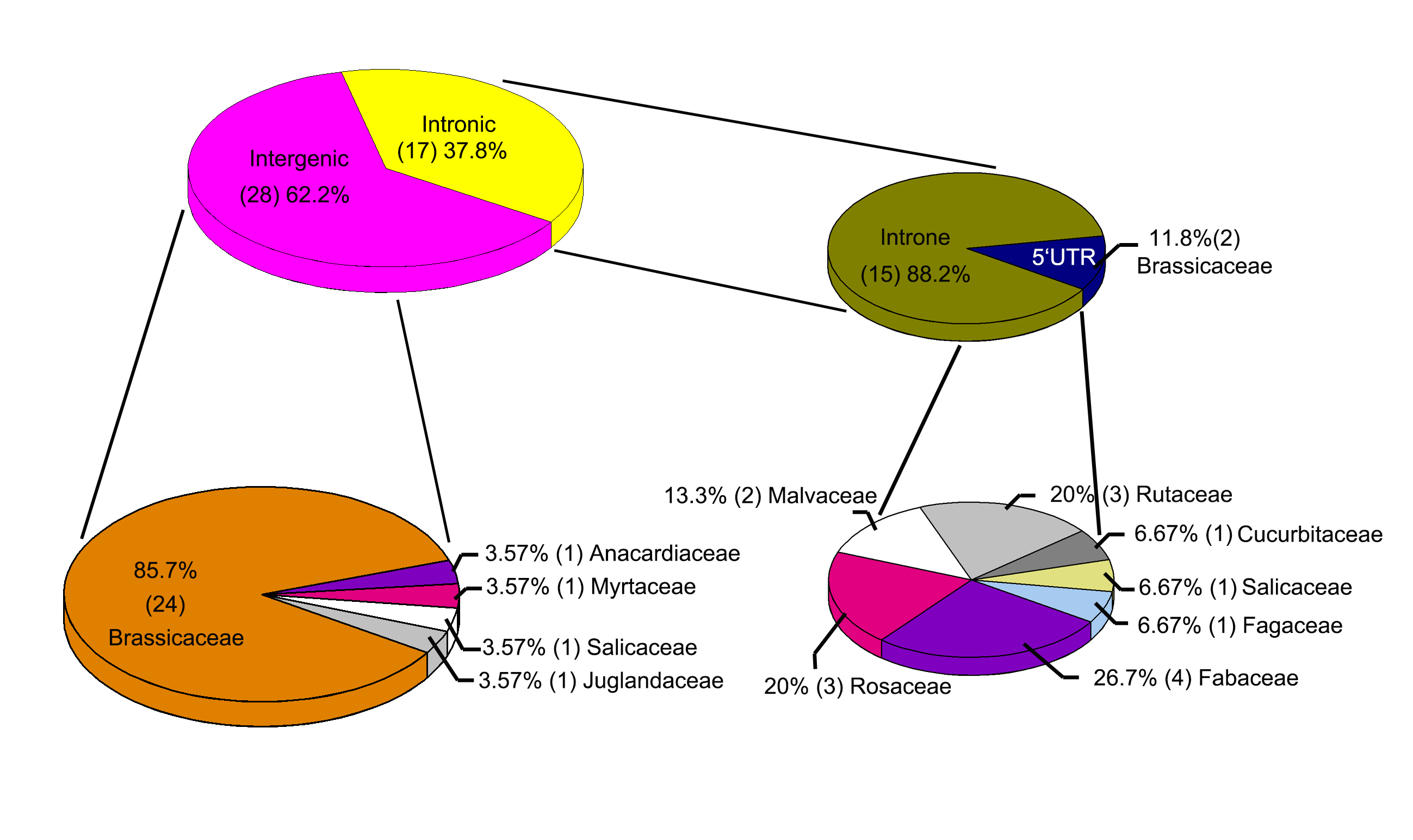


**FIGURE S3 Locations of miR9560s scattered at the intergenic and intronic regions in different species. This is the statistical analysis of FIGURE 1a**


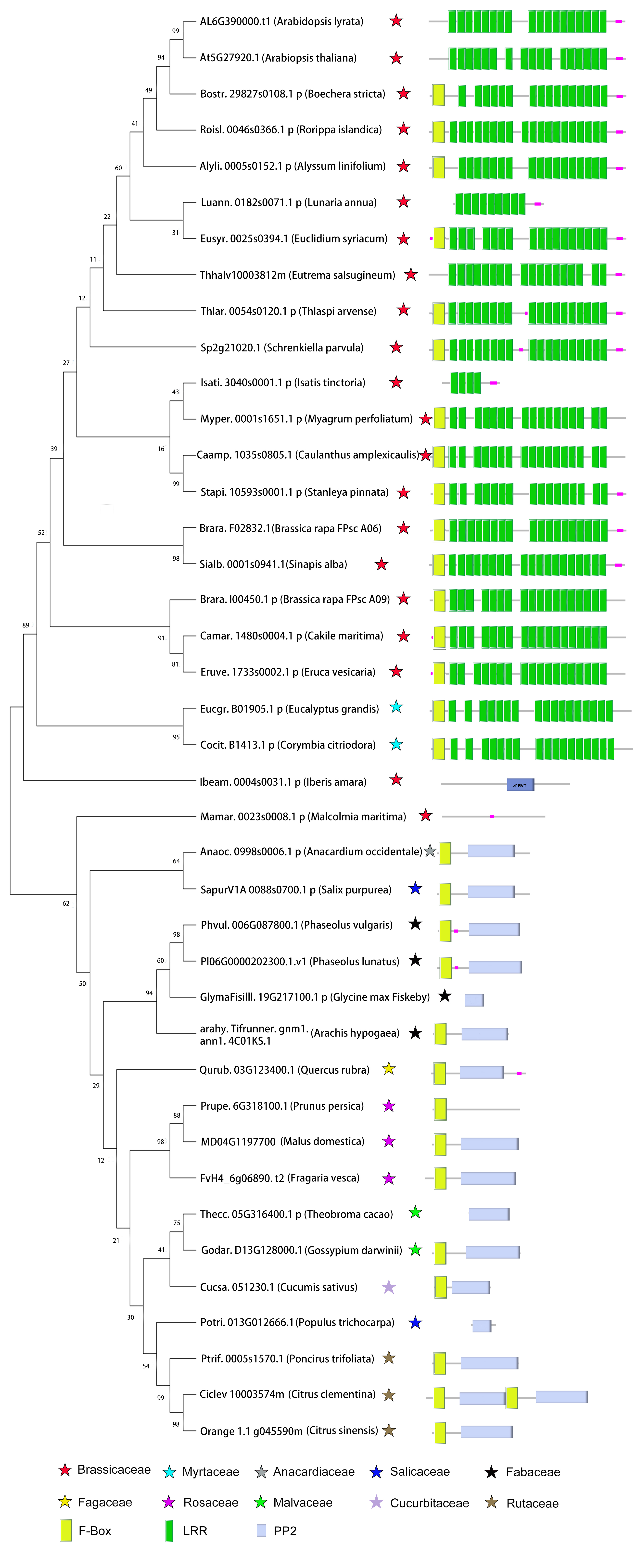


**FIGURE S4** **Phylogenetic analysis of the genes nearby miR9560 in plants**


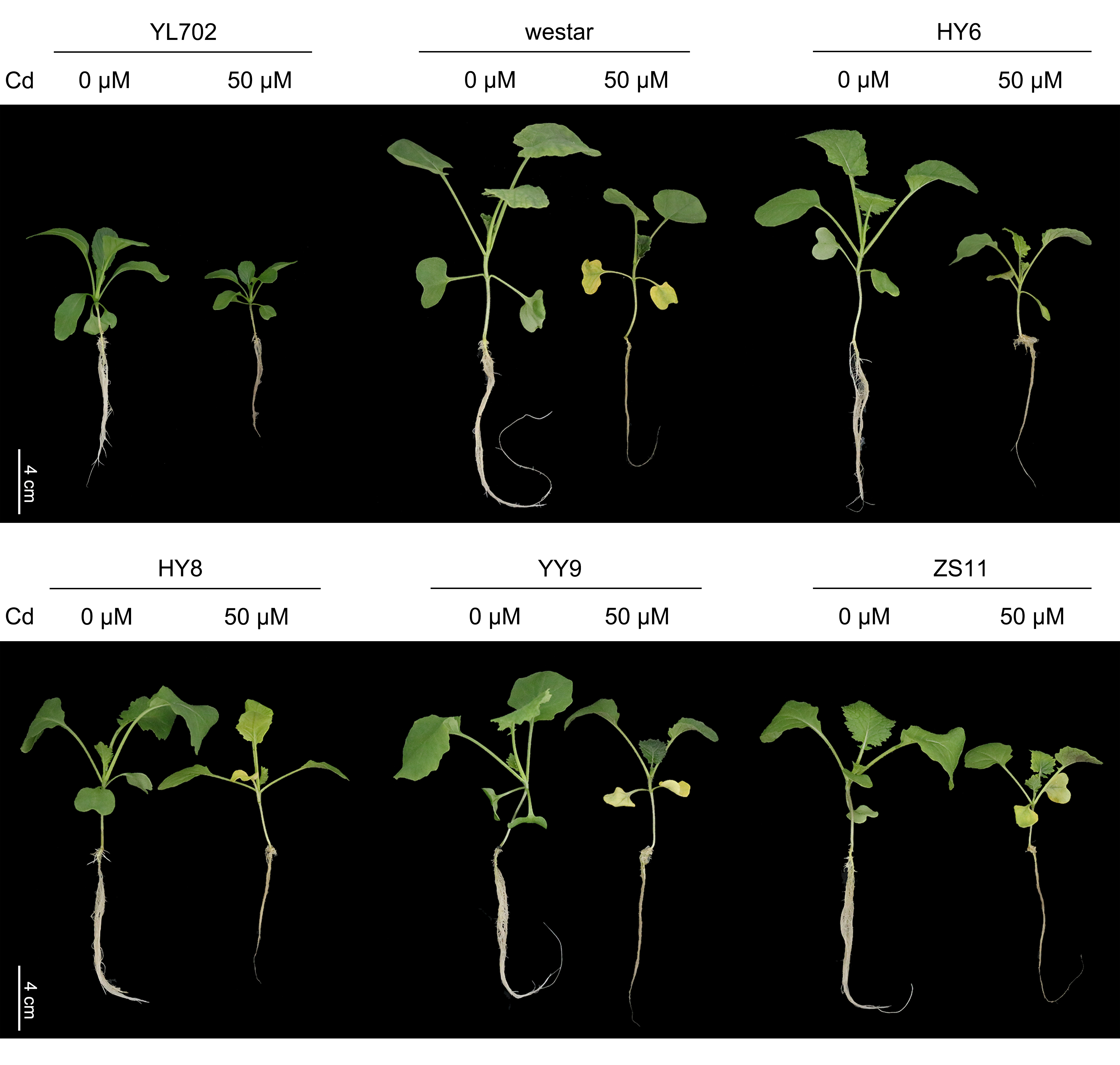


**FIGURE S5 *Brassica* plants treated with or cadmium**

The plants were initially grown in ½ Hoagland solution for 12 days. Subsequently, they were divided into two groups: one group remained as control plants without Cd exposure, while the other group was subjected to 50 µM Cd stress for four days.


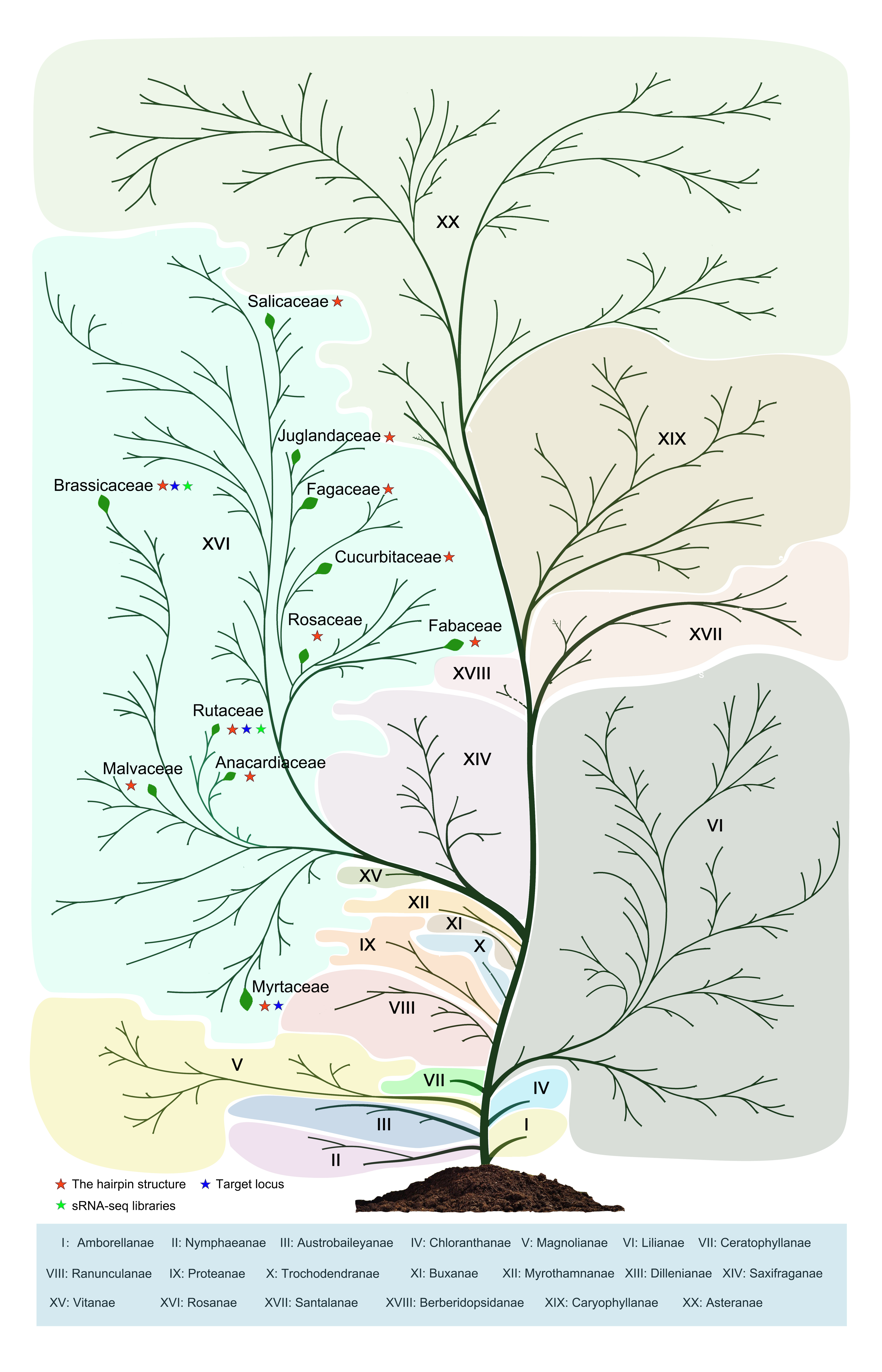


**FIGURE S6 The existence of miR9560 in flowering plants**

Different background color represents different superorder. Branch with green leaf indicates that the secondary structure of miR9560 could be found at least in one species in this family. The red, blue, and green stars respectively indicate that the secondary structure of miR9560, the target locus of miR9560 and the sRNA-seq libraries have been identified in some species of the genus.

**Table S1 Primer sequences used for RNA gel blotting and PCR**

| **Primer Name** | **Primer Sequences (5’-3’)** |
| --- | --- |
| **For Northern Blot** |  |
| miR9560 | ACTCATATTTGTTCCACCACCTGT |
| **For Real Time PCR** |  |
| primiR9560a-qF | GTCAAAAGAGAAAAATGTATCGACT |
| primiR9560a-qR | ccgaggCAGGAATATAAGAATATGTG |
| primiR9560b-qF | cggcGAAGCCAAAGATAATGTACC |
| primiR9560b-qR | gcggcgATATAACACAGGAATATACG |
| **For semi-quantitative PCR** |  |
| pF1-qF | cgcgCGCTAGCTATATATGCCTTTC |
| pF1-qR | gccgCTGTGTTTCGATAAGTCGTTC |
| pF2-qF | cgcGTAGAACGACTTATCGAAACAC |
| pF2-qR | cggGCACACTTCCACTATCTTTCC |
| pF3-qF | CGCCAGTAAAGGACATAAAGC |
| pF3-qR | AACGTGCATTACTGAAGGTGC |

**Table S2 miR9560 in *Arabidopsis thaliana* (Xu *et al.*, 2017)**

| Sample | Repeat | Total mapped reads | miR9560 reads  (one nt variants) | miR9560 TPM |
| --- | --- | --- | --- | --- |
| 48h_silique | rep1 | 5757672 | 218 | 36.07 |
|  | rep2 | 2716183 | 17 | 5.90 |
| 72h_silique | rep1 | 4946434 | 744 | 143.00 |
|  | rep2 | 2973955 | 145 | 46.56 |
| 120h_silique | rep1 | 4006546 | 241 | 56.43 |
|  | rep2 | 3634172 | 53 | 13.91 |
| 7d_silique | rep1 | 5876728 | 442 | 70.26 |
|  | rep2 | 3146135 | 30 | 9.03 |
| Juvenile_leaf | rep1 | 3483514 | 4 | 1.08 |
|  | rep2 | 2318661 | 2 | 0.79 |
| Adult_leaf1 | rep1 | 4054561 | 54 | 12.62 |
|  | rep2 | 1825547 | 0 | 0.00 |
| Adult_leaf2 | rep1 | 3933186 | 34 | 8.30 |
|  | rep2 | 2921145 | 1 | 0.32 |
| Cauline_leaf | rep1 | 3573584 | 44 | 11.19 |
|  | rep2 | 2401632 | 2 | 0.77 |
| Hypocotyl | rep1 | 4774952 | 1 | 0.20 |
|  | rep2 | 3090623 | 0 | 0.00 |
| Cotyledon | rep1 | 4446809 | 0 | 0.00 |
|  | rep2 | 2209034 | 0 | 0.00 |
| Inflorescence | rep1 | 4291730 | 56 | 12.58 |
|  | rep2 | 2490706 | 3 | 1.14 |
| Stem_1st_node | rep1 | 4539774 | 69 | 14.37 |
|  | rep2 | 2958130 | 9 | 2.86 |
| Flower_12_sepals | rep1 | 3820644 | 35 | 8.68 |
|  | rep2 | 2586895 | 1 | 0.36 |
| Flower_12_petals | rep1 | 5073816 | 227 | 42.37 |
|  | rep2 | 2953083 | 63 | 20.13 |
| Flower_12_stamens | rep1 | 5415176 | 95 | 16.31 |
|  | rep2 | 2075684 | 26 | 11.53 |
| Flower_12_carpels | rep1 | 6338401 | 102 | 15.60 |
|  | rep2 | 2959040 | 7 | 2.22 |
| Flower_stage9 | rep1 | 4500000 | 83 | 17.71 |
|  | rep2 | 2307468 | 1 | 0.39 |
| Flower_stage_10_11 | rep1 | 5044446 | 49 | 9.32 |
|  | rep2 | 2034616 | 3 | 1.40 |
| Flower_stage_12 | rep1 | 4238473 | 40 | 8.87 |
|  | rep1-2 | 5748271 | 81 | 13.53 |
|  | rep2 | 2404608 | 1 | 0.39 |
| Full_flower_stage_13 | rep1 | 6286309 | 157 | 24.13 |
|  | rep2 | 3465622 | 1 | 0.28 |
| Flower_13_carpels | rep1 | 6412670 | 127 | 19.15 |
|  | rep2 | 3434954 | 21 | 5.87 |
| Bent_torpedo_embryos | rep1 | 4735242 | 12 | 2.42 |
|  | rep2 | 3427405 | 0 | 0.00 |
| Green_leaf_embryos | rep1 | 4772266 | 15 | 3.03 |
|  | rep1-2 | 5892578 | 4 | 0.65 |
|  | rep1-3 | 5948955 | 1 | 0.16 |
|  | rep2 | 4049313 | 3 | 0.71 |
| Seedling | rep1 | 5742814 | 0 | 0.00 |
|  | rep1-2 | 5611321 | 1 | 0.17 |
|  | rep2 | 2463533 | 0 | 0.00 |
| Root | rep1 | 5157949 | 0 | 0.00 |
|  | rep2 | 3472397 | 0 | 0.00 |
| SAM | rep1 | 3979039 | 0 | 0.00 |
|  | rep2 | 2721068 | 0 | 0.00 |
| Seed | rep1 | 3906605 | 0 | 0.00 |
|  | rep2 | 4259789 | 0 | 0.00 |
